# Identification of non-*Saccharomyces* yeast strains isolated from local traditional sorghum beer produced in Abidjan district (Côte d’Ivoire) and their ability to carry out alcoholic fermentation

**DOI:** 10.1186/s12866-022-02560-8

**Published:** 2022-06-27

**Authors:** Wahauwouélé Hermann Coulibaly, Zamble Bi Irié Abel Boli, Koffi Maïzan Jean-Paul Bouatenin, Ange-Michèle Akissi M’bra, Sonagnon H. S. Kouhounde, Koffi Marcellin Djè

**Affiliations:** 1 Laboratoire de Biotechnologie Et Microbiologie Des Aliments, Unité de Formation Et de Recherche Des Sciences Et Technologie Des Aliments (UFR-STA), Université Nangui Abrogoua, 02 BP 801, Abidjan, Ivory Coast; 2 Laboratoire Des Sciences Biologiques Appliquées, Université Aube Nouvelle, 01 BP 234, Bobo-Dioulasso, Burkina Faso

**Keywords:** *Issatckenkia orientalis*, *Pichia kudriavzevii*, Non-*Saccharomyces*, Traditional sorghum beer

## Abstract

Studies on yeasts involved in traditional sorghum beer fermentation in several African countries revealed the presence of two groups: *Saccharomyces* and non-*Saccharomyces*. If *Saccharomyces* strains were reputed for their fermentation performances, the non-*Saccharomyces* yeast strains have been recognized for their contribution towards the improvement of the beverage's organoleptic quality, justifying their use as aromatic starter. In spite of this contribution, most studies were focused only on *Saccharomyces* strains as starter. In this work, the non-*Saccharomyces* yeast strains found in the inoculum of traditional sorghum beer was investigated. Identification of non-*Saccharomyces* yeast strains by PCR-RLFP followed sequencing of D1/D2 domain revealed the presence of 2 species: *Issatckenkia orientalis* and *Pichia kudriavzevii* with *Issatckenkia orientalis* predominating. Out of the two species, fermentation characteristics showed that *Issatckenkia orientalis* seemed suitable for sorghum beer production.

**Supplementary Information:**

The online version contains supplementary material available at 10.1186/s12866-022-02560-8.

## Introduction

Sorghum (*Sorghum bicolor (L.) Moench*) plays an important role in food security in developing countries. Generally, sorghum grains are used in the preparation of many local foods such as breads, pasta, porridge and pancakes. In many sub-Saharan African countries where sorghum is cultivated, it is used in the preparation of traditional beers, commonly named sorghum beers or traditional sorghum beers or opaque beers. The name of beer varied according to geographical location where it is produced, thus pito or burukutu in Nigeria, chibuku in Zimbabwe, dolo in Mali and Burkina Faso, bili-bili in Chad, tchapalo in Côte d’Ivoire [1–3].

The production and commercialization of this beer have socio-economic importance because it is consumed during various festivals and traditional ceremonies (e.g., marriage, birth, baptism, the handing over of a dowry, etc.) and constitute a source of important income for women who produce it [4]. However, despite its socio-economic importance, the production of tchapalo as the others traditional beverages is rudimentary and resulting in products with the low quality [5]. During fermentation, aromatic compounds production is strongly determined through microbial metabolism. By virtue of their volatile compounds production, those microorganisms are essential to the final quality of product. Like other traditional beverages, from a sensory point of view traditional sorghum beer is one of the most instable [6]. The recourse to starter culture was thus recommended for resolving the microbiological and organoleptic instability of the beverage. [5–7]. The vast majority of starters applied focused on reducing fermentation time, ethanol resistance of selected strains, fermentation control and microbiological stability [4, 8–15]. During the brewing of traditional sorghum beer, several authors reported that the alcoholic fermentation was carried out predominantly by *Saccharomyces cerevisiae*. The presence of others yeasts species, the non-*Saccharomyces* has been reported in beers from from varied geographic origins. Sefa-Deheh et al. [8]; Lyumugabe et al. [14]; N'guessan et al. [16] reported *Candida tropicalis* as the predominant non-*Saccharomyces* yeast in pito and tchapalo (traditional sorghum beers of Ghana and Côte d’Ivoire respectively), while *Clavispora lusitaniae* and *Candida inconspicua* have been identified from tchoukoutou (traditional sorghum beer of Benin) and ikigage (traditional sorghum to Rwanda) respectively, as the predominant non-*Saccharomyces.* [12, 17]. Earlier studies regarding the application of starter cultures for traditional sorghum beer production focused on the use of *S. cerevisiae*, having been identified as the dominant specie [7, 12]. In this last decade, there is a growing use of non-*Saccharomyces* yeasts species (*Brettanomyces bruxellensis, Torulaspora delbrueckii, Candida shehatae, Candida tropicalis, Zygosaccharomyces rouxii, Lachancea thermotolerans, Saccharomycodes ludwigii, and Pichia kluyveri* for their flavor profile in association with *Saccharomyces cerevisiae* in mixed culture suggesting a large contribution to beer organoleptic quality [18–24]. The aroma instability of sorghum beers makes them less attractive than industrial beers. In order to offer consumers a product of consistent quality with attractive flavors, it would be useful to introduce a starter based on the flavor profile of non-*Saccharomyces* yeast. In this study we investigated the alcoholic fermentation performance of identified and selected non-*Saccharomyces* yeasts in sorghum wort.

## Results

### Sorghum wort pasteurized characteristics

The physicochemical characteristics of sorghum wort used to carry out the alcoholic fermentation with wild yeasts (traditional inoculum) are given in Table 1. Thus, pH value was 3.53 ± 0.005 and the titratable acidity was 0.495 ± 0.01%. Total soluble solids value of 11.7 ± 0.14°Brix and the density was 1.011 ± 0.001. The sorghum wort was free of alcohol (0% v/v).

**Table 1:** Physicochemical characteristics of sorghum wort pasteurized

	Sorghum wort
Density	1.041 ± 0.001
pH	3.53 ± 0.005
Titratable acidity (%)	0.49 ± 0.01
Total Soluble Solids (°Brix)	11.7 ± 0.14
Alcohol rate (% v/v)	0 ± 0

### Changes of pH and titratable acidity (TA) during alcoholic fermentation

**Fig. 1:**
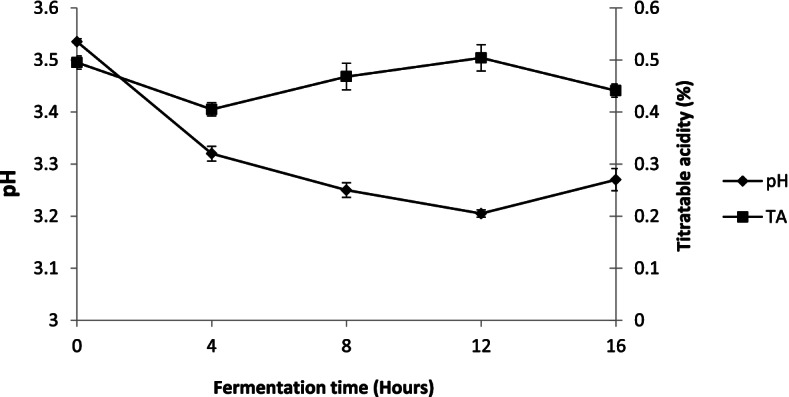
Changes of pH and TA during alcoholic fermentation sorghum wort

Figure 1 shows pH and titratable acidity changes during alcoholic fermentation. Three phases of development were observed for each parameter. The first phase, from 0 to 4 h of fermentation, was characterized by a decreasing in pH and TA values from 3.53 ± 0.005 to 3.32 ± 0.01 and from 0.495 ± 0.01 to 0.405 ± 0.01% respectively. The second phase ranged between 4 and 12 h, where pH values decreased while those of TA values increased. pH values decreased from 3.32 ± 0.01 to 3.2 ± 0.007 followed by an increase in TA from 0.405 ± 0.01 to 0.504 ± 0.02%. In contrast to second phase, last phase which extended from 12 to 16 h (end fermentation) was characterized by a slight increase in pH (3.2 ± 0.007 to 3.27 ± 0.02) and a decrease in TA (0.504 ± 0.02 to 0.441 ± 0.01).

### Changes of total soluble solids (TSS) and alcohol level during alcoholic fermentation of sorghum wort

**Fig. 2:**
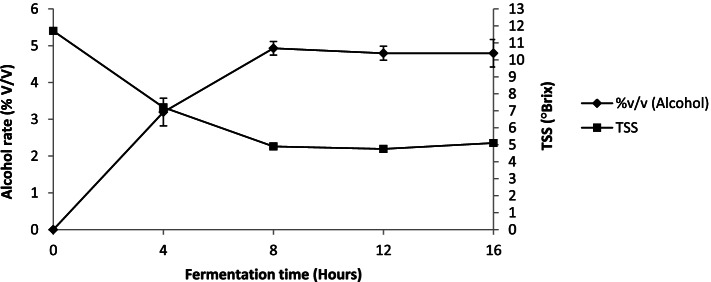
Changes of TSS and alcohol rate during alcoholic fermentation sorghum

The TSS and alcohol level evolution during alcoholic fermentation were contrary during the first half of the fermentation time (8 h) (Fig. 2). During this period, decreasing of TSS values was simultaneously with alcohol rate increasing from 11.7 ± 0.14 to 4.9 ± 0.14°Brix and from 0 ± 0 to 4.93 ± 0.18% (v/v) respectively. From 8 h of fermentation, TSS and alcohol values tend to stabilize with slight variation observed. TSS values increase from 4.9 ± 0.14 to 5.1 ± 0.14°Brix and those of alcohol content from 4.93 ± 0.18 to 4.79 ± 0.3% (v/v).

### Microbial load development during alcoholic fermentation of sorghum wort

**Fig. 3:**
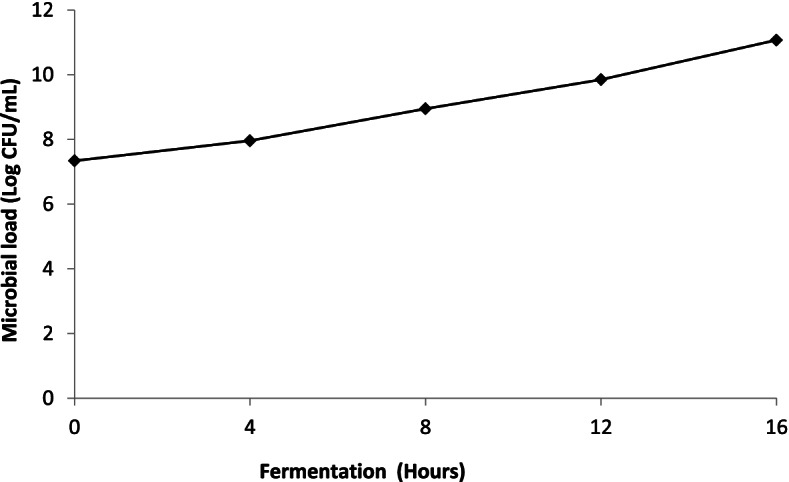
Microbial load evolution during alcoholic fermentation sorghum wort

A continuous increase in the microbial load was observed during the course of alcoholic fermentation (Fig. 3). This increase in microbial load was less significant during the first half of fermentation (from 7.34 to 8.95 log CFU/mL). On the other hand, the second half of the fermentation time was characterized by a greater increase in the microbial load (from 8.95 to 11.07 log CFU/mL).

### Dynamic of non-*Saccharomyces* yeast species

#### Non-*Saccharomyces* yeast species identification during alcoholic fermentation

Amplification of the 5.8S-ITS region of the rDNA after agarose gel electrophoresis was positive for all 30 presumptive NS species isolates. The amplicons generated by the 30 isolates were all the same size, around 500 bp (Table 2). Amplicons enzymatic hydrolysis has allowed obtaining 3 groups. The Group I was consisted of two restriction fragments after digestion with *Hae III* (390 bp and 90 bp), and two fragments after digestion with *Hinf I* (270 bp and 220 bp). The group II restriction fragments after *Hae III* digestion yielded a single 500 bp fragment and two fragments (270 bp and 220 bp) after *Hinf I* digestion. In the last group (III), the fragment digested by *Hae III* as well as the one digested by *Hinf I* had a size of 500 bp (Table 2). The identified strains belonged to the species *Issatchenkia orientalis* and *Pichia kudriavzevii* according to sequencing results with 99.48%-100% homology to the GenBank sequences. The isolates from start fermentation until 8 h belonged to the *Issatchenkia orientalis* (Group I) and those from end fermentation have been identified as *Pichia kudriavzevii* (Group II and III) (Table 3). The partial sequences of the all yeast strains were deposited in the NCBI database (the accession numbers are listed in Table 3).

**Table 2:**
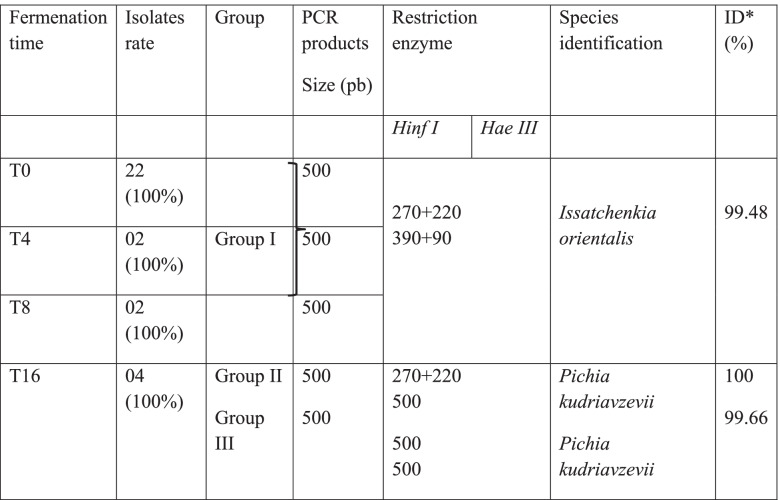
Non-*Saccharomyces* species identity on the basis of their lengths of restriction fragments of the 5.8S-ITS rDNA gene region and the highest D1/D2 domain similarity score and rate of yeast strains (%) isolated from different stages of fermentation

*ID*: percentage of identification, * According to BlastN search of 5.8S-ITS rDNA gene sequences in NCBI database

**Table 3:** Nucleotide sequences of yeast species

Group	Nucleotide sequence	Specie	Accession no
Group I	ACAAAGGGGATGCCTCAGTAGCGGCGAGTGAAGCGGCAAGAGCTCAGATTTGAAATCGTGCTTTGCGGCACGAGTTGTAGATTGCAGGTTGGAGTCTGTGTGGAAGGCGGTGTCCAAGTCCCTTGGAACAGGGCGCCCAGGAGGGTGAGAGCCCCGTGGGATGCCGGCGGAAGCAGTGAGGCCCTTCTGACGAGTCGAGTTGTTTGGGAATGCAGCTCCAAGCGGGTGGTAAATTCCATCTAAGGCTAAATACTGGCGAGAGACCGATAGCGAACAAGTACTGTGAAGGAAAGATGAAAAGCACTTTGAAAAGAGAGTGAAACAGCACGTGAAATTGTTGAAAGGGAAGGGTATTGCGCCCGACATGGGGATTGCGCACCGCTGCCTCTCGTGGGCGGCGCTCTGGGCTTTCCCTGGGCCAGCATCGGTTCTTGCTGCAGGAGAAGGGGTTCTGGAACGTGGCTCTTCGGAGTGTTATAGCCAGGGCCAGATGCTGCGTGCGGGGACCGAGGACTGCGGCCGTGTAGGTCACGGATGCTGGCAGAACGGCGCAACACCGCCCGTCTTGAAACCCGGGAACAAAAAA	*Issatchenkia orientalis*	ON229495
Group II	ACAAAGGGATGCCTCAGTAGCGGCGAGTGAAGCGGCAAGAGCTCAGATTTGAAATCGTGCTTTGCGGCACGAGTTGTAGATTGCAGGTTGGAGTCTGTGTGGAAGGCGGTGTCCAAGTCCCTTGGAACAGGGCGCCCAGGAGGGTGAGAGCCCCGTGGGATGCCGGCGGAAGCAGTGAGGCCCTTCTGACGAGTCGAGTTGTTTGGGAATGCAGCTCCAAGCGGGTGGTAAATTCCATCTAAGGCTAAATACTGGCGAGAGACCGATAGCGAACAAGTACTGTGAAGGAAAGATGAAAAGCACTTTGAAAAGAGAGTGAAACAGCACGTGAAATTGTTGAAAGGGAAGGGTATTGCGCCCGACATGGGGATTGCGCACCGCTGCCTCTCGTGGGCGGCGCTCTGGGCTTTCCCTGGGCCAGCATCGGTTCTTGCTGCAGGAGAAGGGGTTCTGGAACGTGGCTCTTCGGAGTGTTATAGCCAGGGCCAGATGCTGCGTGCGGGGACCGAGGACTGCGGCCGTGTAGGTCACGGATGCTGGCAGAACGGCGCAACACCGCCCGTCTAAAACCCGGGGAACAAA	*Pichia kudriavzevii*	ON229496
Group III	AACAAAGGGATGCCTCAGTAGCGGCGAGTGAAGCGGCAAGAGCTCAGATTTGAAATCGTGCTTTGCGGCACGAGTTGTAGATTGCAGGTTGGAGTCTGTGTGGAAGGCGGTGTCCAAGTCCCTTGGAACAGGGCGCCCAGGAGGGTGAGAGCCCCGTGGGATGCCGGCGGAAGCAGTGAGGCCCTTCTGACGAGTCGAGTTGTTTGGGAATGCAGCTCCAAGCGGGTGGTAAATTCCATCTAAGGCTAAATACTGGCGAGAGACCGATAGCGAACAAGTACTGTGAAGGAAAGATGAAAAGCACTTTGAAAAGAGAGTGAAACAGCACGTGAAATTGTTGAAAGGGAAGGGTATTGCGCCCGACATGGGGATTGCGCACCGCTGCCTCTCGTGGGCGGCGCTCTGGGCTTTCCCTGGGCCAGCATCGGTTCTTGCTGCAGGAGAAGGGGTTCTGGAACGTGGCTCTTCGGAGTGTTATAGCCAGGGCCAGATGCTGCGTGCGGGGACCGAGGACTGCGGCCGTGTAGGTCACGGATGCTGGCAGAACGGCGCAACACCGCCCGTCTTGAACCACACGGACCAAAACC	*Pichia kudriavzevii*	ON229497

**Fig. 4:**
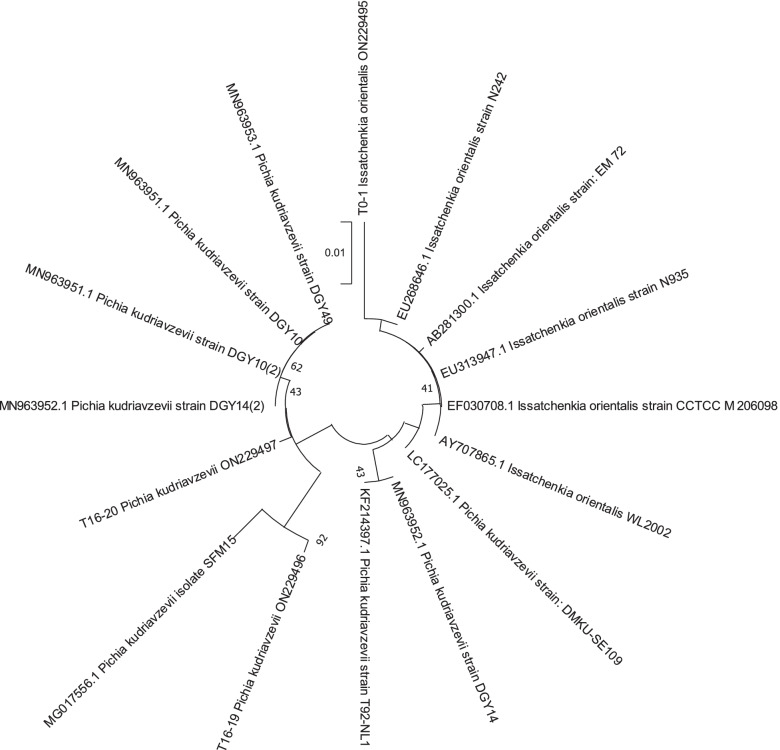
Phylogenetic tree of Non-*Saccharomyces* strains

The phylogenetic tree revealed the existence of family relationship between the strains in this study and the those of database of Genbank (Fig. 4). *Issatchenkia orientalis* ON 229,495 strain T0-1 was related to *Issatchenkia orientalis* EU 264,686.1 strain N242. *Pichia kudriavzevii* ON 229,496 strain T16-19 was related to *Pichia kudriavzevii* MG 017,556.1 isolate SFM 15. *Pichia kudriavzevii* ON 229,497 strain T16-20 was related to *Pichia kudriavzevii* MN963952.1 strain DGY14, *Pichia kudriavzevii* MN963951.1 strain DGY10 and *Pichia kudriavzevii* MN963953.1 strain DGY49.

### Characteristics fermentation of Issatchenkia orientalis and Pichia kudriavzevii

#### Growth of *Issatchenkia orientalis* and *Pichia kudriavzevii* at differents pH, Temperature (°C) and Ethanol rate (%)

**Fig. 5:**
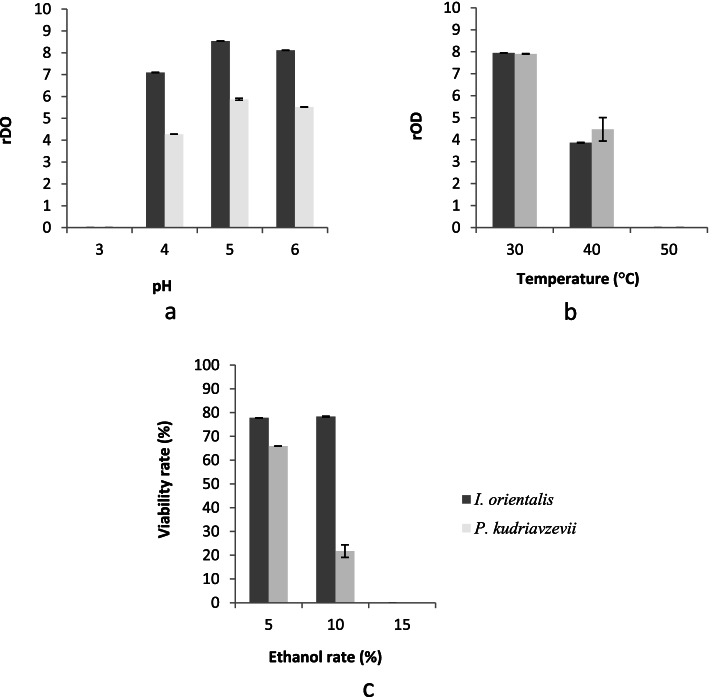
Growth of *Issatchenkia orientalis* and *Pichia kudriavzevii* at differents pH (5**a**), at differents temperatures (°C) (5**b**) and at differents ethanol rates (%) (5**c**)

Figure 5 showed the growth of *Issatchenkia orientalis* and *Pichia kudriavzevii* at differents pH, Temperature (°C) and Ethanol rate (%).

Growth test at differents pH revealed that generally, *Issatchenkia orientalis* specie showed a higher growth rate than *Pichia kudriavzevii* specie except at pH 3 where there was no growth. (Fig. 5a). Furthermore, whatever the pH tested, the OD ratio values were statistically different (*P* < 0.05). For the two species tested, the pH range for growth was between pH 4 and pH 6 with an optimum growth observed at pH 5 (optimum pH).

The growth test at different temperature showed two species had the same level of growth at 30 °C. The ration of OD values were 7.94 ± 0.007 and 7.9 ± 0.014 respectively *Issatchenkia orientalis* and *Pichia kudriavzevii*. Statistical analyses showed no significant difference (*P* > 0.05) in OD ratio between the 2 species (Fig. 5b) at this temperature. In contrast, at 40 °C, the growth of *Pichia kudriavzevii* specie was higher than *Issatchenkia orientalis* specie. Ratio O.D values were 4.47 ± 0.53 for *Pichia kudriavzevii* specie, while this for *Issatchenkia orientalis* was 3.83 ± 0.014. Statistical analyses showed a significant difference (*P* < 0.05) in OD ratio between the two species. None Non-*Saccharomyces* of the species were able to grow at 50 °C.

Among the two non-*Saccharomyces* strains identified in this study, *Issatchenkia orientalis* exhibited a relatively higher resistance to 5% and 10% (v/v) ethanol than *Pichia kudriavzevii* (Fig. 5c). At 5% (v/v) ethanol, the viability of *Issatchenkia orientalis* was 77.76 ± 0.1% compared to 65.9 ± 0.07% for *Pichia kudriavzevii*. *Issatchenkia orientalis* specie recorded a viability rate of 78.32 ± 0.17% while that *Pichia kudriavzevii* specie was 21.74 ± 2.65% at 10% (v/v) ethanol level. Statistical analysis showed a significant difference (*P* < 0.05) in viability rate between the 2 species. The 15% (v/v) ethanol rate was fatal for both species, as no growth occurred. From tests carried out, *Issatchenkia orientalis* specie showed the best ability to carry out fermentation than *Pichia kudriavzevii* specie and was therefore chosen for use in the continuing part of the study.

### Physicochemical characteristics and organic acids content of sorghum wort and the produced beers

#### Physicochemical characteristics of sorghum wort and the produced beers (beer from *Issatchenkia orientalis* and beer from *Saccharomyces cerevisiae*)

The physicochemical characteristics of sorghum wort and the produced beers (beer from *Issatchenkia orientalis:* BIo and beer from *Saccharomyces cerevisiae:* BSc) are shown in Table 4. For all physicochemical characteristics of the two beers, statistical analyses showed significant difference between them. Specific gravity, pH and TSS values of the beer BIo were higher than those of beer BSc. These values were 1.009 ± 0.0014; 3.11 ± 0.01 and 9.5 ± 0.1° Brix for specific gravity, pH and ESR respectively for beer Blo. In contrast, the TA and beer alcohol content (BSc) were higher with 1.24 ± 0.01% and 5.46% (v/v) respectively.

**Table 4:** Physicochemical characteristics of sorghum wort and beers (Bio and BSc)

	Sorghum wort	BSc	BIo
Specific gravity	1.044 ± 0^a^	1.003 ± 0.001^b^	1.009 ± 0.001^c^
pH	3.22 ± 0^a^	3.06 ± 0.007^b^	3.11 ± 0.01^c^
TA (%)	0.751 ± 0.019^a^	1.24 ± 0.01^b^	1.08 ± 0.01^c^
Total Solids Soluble (° Brix)	15.15 ± 0.07^a^	8.7 ± 0.1^b^	9.5 ± 0.1^c^
Alcool rate (%v/v)	0 ± 0^a^	5.46 ± 0.19^b^	4.66 ± 0.19^c^

The values expressed are the means of three measurements. On the same line, the mean values with the same letter are not significantly different (*p* > 0.05)

#### Organic acids content of sorghum wort and the produced beers

Table 5 showed the organic acids content of wort and beers fermented by *Issatchenkia orientalis* and *Saccharomyces cerevisiae*. Excepted citric acid content, all other organic acids of beer from *Issatchenkia orientalis:* Bio were similar to those of the beer from *Saccharomyces cerevisiae:* BSc. Citric acid was not found in beer obtained *Issatchenkia orientalis* specie, while it was 32.1 ± 1.8 (mg/L) for beer fermented by *Saccharomyces cerevisiae*. For the wort and beer samples (BIo), the highest concentration was observed for tartaric acid with 23.3 ± 1.4 and 26.5 ± 1.6 g/L for wort and beer (BIo) respectively. Citric acid with 32.1 ± 1.8 mg/L was the most abundant in beer (BSc). The lowest concentrations were recorded for fumaric acid with an identical value of 0.246 mg/L for the wort and the 2 beers (BSc and BIo). On the hand-over, *Issatchenkia orientalis* and *Saccharomyces cerevisiae* have not influenced tannic acid and fumaric acid content comparatively to wort. Statistical analyses showed no significant difference (*P* > 0.05) between the samples, excepted for citric acid. The latter was not detected in the wort as in BIo beer, but in beer produced from *S.* *cerevisiae*.

**Table 5:** Organic acids content in sorghum wort and beers (Bio and BSc)

	Sorghum wort	BSc	BIo
Tartric acid (g/L)	23.3 ± 1.4^a^	23.8 ± 1.2^a^	26.5 ± 1.6^a^
Lactic acid (g/L)	21.85 ± 0.7^a^	18 ± 1.1^a^	18.15 ± 1,56^a^
Citric acid (mg/L)	0^a^	32.1 ± 1.8^b^	0^a^
Acetic acid (mg/L)	7.15 ± 0.9^a^	7 ± 0.7^a^	7.15 ± 1.01^a^
Tannic acid (mg/L)	1.76 ± 0^a^	1.76 ± 0^a^	1.76 ± 0^a^
Fumaric acid (mg/L)	0.246 ± 0^a^	0.246 ± 0^a^	0.246 ± 0^a^

The values expressed are the means of three measurements. On the same line, the mean values with the same letter are not significantly different (*p* > 0.05)

## Discussion

Non-*Saccharomyces* yeast strains have been considered for a long time as harmful microorganisms, but growing interest in their application in alcoholic fermentations has led to many studies on them in recent years.

Some studies aimed at aromatic contribution to beer [18–24], and some others on their antimicrobial activities through the production of killer toxins which inhibit of growth of undesirable microorganisms as in the example of *T. delbrueckii* and *Wickerhamomyces anomalus* [25]. Non-*Saccharomyces* yeasts have been increasingly considered in the production of beer [26]. The identification yeast isolates during alcoholic fermentation of sorghum wort had revealed occurence of 2 species for all isolates identified: *Issatchenkia orientalis* and *Pichia kudriavzevii*. The low ratio of NS found in this work is in line with the findings of Maoura et al. [10]; Lyumugabe et al. [14]; N’Guessan et al. [16] who also reported the predominance of *Saccharomyces cerevisiae* strains in spontaneously fermented sorghum beer. The *S. cerevisiae* strain population were 55–90%, 92.84% and 99% in the sorghum beer produced in Ghana, Burkina-Faso and Côte d’Ivoire. The types and levels of presence of NS species is also dependent on the location of beer preparation. Maoura et al. [10] identified *Kluyveromyces maxianus* as the major NS specie in traditional sorghum beer (bili bili) produced in Chad. The specie *Issatckenkia orientalis* was identified by Lyumugabe et al. [16] as the major NS specie in Ikigage, a traditional sorghum-based beer produced in Rwanda. *Issatckenkia orientalis* has been identified as the major NS specie in this present study. Lyumugabe et al. [10] also used *Issatckenkia orientalis* specie in monoculture and mixed culture with *Saccharomyces cerevisiae* to improve the aromatic quality of traditional sorghum beer Ikigage produced in Rwanda. In Côte d’Ivoire, *Candida tropicalis* associated with *Saccharomyces cerevisiae* has been tested to enhance the organoleptic quality of tchapalo, traditional sorghum beer [6]. Other investigators, such as Konlani et al. [8]; Sefa-Dedeh et al. [27] reported the presence of *C. tropicalis, P. kudriavzevii, Kloeckera apiculata, Pichia anomala, Torulaspora delbrueckii, Schizosaccharomyces pombe, Kluyveromyces africanus* as NS species in traditional sorghum beers.

According to Jespersen, [28]; van der Aa Kühle et al. [29] distribution of yeast populations involved in alcoholic fermentation of traditional African sorghum beer is strongly influenced by regional area, differences between processing steps and ingredients used.

Population distribution in this study showed NS yeasts occurence throughout the alcoholic fermentation.Essentially, the role of the NS species is to enchance organoleptic quality of fermented products through contribution to aromatic complexity [30, 31]. In their studies Ciani et al. [24]; Methner et al. [32] reported that some NS species, such as *Torulaspora delbrueckii, Cyberlindnera saturnus, Kluyveromyces marxianus, Saccharomycopsis fibuligera,* are used in monocultures or combined with *S. cerevisiae*, to enchance aromatisation of beer. Association of the species of *Saccharomyces* and non-*Saccharomyces* in sorghum wort fermentation may have beneficial aspects such as inhibition of some species. Indeed, Halm and Olsen [33] reported that combination of *S. cerevisiae* and *P. kudriavzevii* in fermentation inhibited the growth of moulds such as *Penicillium citrinum*, *Aspergillus flavus*, *Aspergillus parasiticus* which produce mycotoxin.

The study of fermentation characteristics (pH, temperature and tolerance to ethanol) of the two species identified *Issatchenkia orientalis* and *Pichia kudriavzevii* showed that *Issatchenkia orientalis* specie was most suitable to carry out the fermentation on account of its superior viability rate than *Pichia kudriavzevii*. According to Aldiguier [34] these parameters are very important to conduct alcoholic fermentation. *Issatchenkia orientalis* specie has been used in several studies to improve beverages quality. Few studies were focused on aroma compounds of tradional sorghum beer. Only, Coulibaly et al. [6], N’Guessan et al. [14] and Lyumugabe et al. [35] have done. It has been reported that use *Issatchenkia orientalis* specie in association with *S. cerevisiae* and *L. fermentum* improved Ikigage quality, traditional sorghum beer to Rwanda. Specifically, *I. orientalis* alone contribuated to butyric acid and 2-phenylethanol production in Ikigage [35]. In according, to Durfour et al. [36] the 2-phenylethanol was among higher alcohols which have an important role in beer flavour. For these authors, to render ikigage beer with homogeneous organoleptic qualities and a high ethanol level, the combination of *S. cerevisiae* with *L. fermentum* and *I. orientalis* as stater cultures is recommended. This procedure also decreases the possibility of food safety concerns in the brew and also possibly will improve the keeping quality of ikigage beer. Isono et al. [37] showed that *I. orientalis* MF-121 was able to produce a significant amount of ethanol under stress conditions. They also reported that malic acid was reduced to 0.33 mg/ml in co-fermentation using a 1:1 (v/v) inoculum ratio of *S. cerevisiae* W-3 and *I. orientalis* KMBL 5774, compared to 1.1 mg/ml with *S. cerevisiae* W-3 alone [38]. On the hand-over, the beer produced from *S. cerevisiae* BSc was more acidified than this produced by *I. orientalis*.

Regarding *Pichia kudriavzevii*, Dos Santos et al. [39] reported that two brazilian native yeast strains influenced the craft beer bioaromatization, improving the acceptance test score with the tasters. Also these *Pichia kudriavzevii* strains were able to produce 28 volatils compounds mainly 20 esters, 2 alcohols, 5 carboxylic acids and 1 hydrocarbon.

Also, Ghosh et al. [40] showed that indigenous strains *Pichia kudriavzevii* isolated and identified from traditional rice beer «Gora» in India, were could improved quality beer in respect of taste, nutritional requirements than industrial strain.

## Conclusion

The study of identification of NS specie contained in traditional inoculum revealed the occurrence of 2 species: *Issatckenkia orientalis* and *Pichia kudriavzevii* with predominance of *Issatchenkia orientalis*. From the two species identified, *Issatchenkia orientalis* was found to be more suitable to carry out alcoholic fermentation. The two beers produced from them were characterized by cereal note and sour taste with high score of preference for beer from *Issatchenkia orientalis*. It is recommended that in further study, the aromatic profile of *Issatckenkia orientalis* should be investigated for its use as aromatic starter in monoculture or mixed culture with *Saccharomyces cerevisiae*.

## Materials and methods

### Yeast strains and sorghum wort

The wild yeasts (traditional inoculum) and sorghum wort used in this study have been collected from randomly selected commercial traditional sorghum beer brewers at Williamsville-Macaci in the District of Abidjan (Southern Côte d’Ivoire). The slurry of wild yeasts was been sun dried into brown powder and lumps.

### Fermentation conditions

Fermentation was carried out under agitation in rotary shaker 1-L sterile flasks fitted with a shaker. The flasks were filled with 500 mL sorghum wort which was previously pasteurized at 100 °C for 10 min and cooled to ambient temperature and closed with dense cotton plugs. Five (05) grams of wild yeasts which correspond to an inoculation rate of 1% (2.10^7^ CFU/mL) were added to the wort. Fermentation was carried out during 24 h at 30 °C with agitator speed of 120 rpm. During latter, samples were collected to 0, 4, 8 and 24 h for physicochemical, microbiology and biology molecular analyses. Three independent experiments were carried out.

### Analytical determination

#### pH, titratable acidity, density and total soluble solids

pH values were determined using a pH-meter (Hanna Instruments; HI 8010) and titratable acidity was measured by titration with 0.1 N NaOH and expressed as % meq of lactic acid in 10 mL of sample respectively. A densimeter (Mettler Toledo) was used to determine the density of each beer in 100 mL of sample and hand refractometer was used to determine total soluble solids (TSS) content expressed as °Brix from two or three drops of sample. For each parameter, three independent measurements were made.

#### Alcohol level determination by specific gravity method

The alcohol level was determined from original gravity (OG) (Density of sorghum wort) and final gravity (FG) (Density of beer) measured using a densimeter as previously described and expressed as follows:

$$Alcohol rate (\mathrm{\% }v/v)=\frac{OG-OF}{0.0075} (1)$$

### Microbiological analysis

#### Isolation and enumeration of yeasts

After decimal dilutions in the YPD medium (10 g/L yeasts extract (Difco)); (10 g/L bactopeptone (Becton Dickinson)); (10 g/L D-glucose (Sordalab)), counts were carried out in duplicate on YPDA (10 g/L yeasts extract (Difco)); 10 g/L bactopeptone (Becton Dickinson)); 10 g/L D-glucose (Sordalab)); Agar 10 g/L (Oxford) medium. The incubation period was two days at 30 °C. The results of the counts were expressed as log (CFU/mL) of fermenting wort and beer.

#### Isolation of non-*Saccharomyces* yeast strains

After enumeration on YPDA, 30 colonies were selected at each sampling time on the basis of size and appearance of colony outline out of a total of 300 colonies. This selection had been repeated two times (biological repetition). The selected colonies were plated on lysine agar medium (Oxoid LTD., Hampshire, England), a selective medium used for to differentiate between non-*Saccharomyces* and *S. cerevisiae* strains. The *Saccharomyces cerevisiae* NRRL Y-12632 strain has been used as negative control. This medium does not allow the growth of *S. cerevisiae* because these species are unable to use lysine for their growth. Isolates where growth was observed were retained for identification by PCR-RLFP followed sequencing.

### Molecular identification of non-*Saccharomyces* yeast isolates

#### Amplification of the ITS region

PCR reaction method performed in this work has been described by Jeyaram et al. [41] on colonies with some modifications. Fresh yeast colonies (less than 5 days old) grown on Sabouraud Chloramphenicol agar were transferred in to 100 mL of molecular biology water under sterility conditions. For the amplification of the 5.8S rDNA-ITS region, the primers ITS1 (5' TCCGTAGGTGAACCTGCGG-3') and ITS4 (5'-TCCTCCGCTTATTGATA-3') were used as described by White et al. [42]. Amplification reactions of the 5.8S rDNA-ITS region were performed using a thermal cycler (Techne Prime). Thermocycler (Techne Prime, UK) according to the program described by Aa Kühle et al. [29] with some modifications:

-First step: Initial denaturation at 95 °C for 10 min.

-Second step: denaturation at 94 °C for 2 min followed by primers annealing at 55.8 °C for 1 min and extension at 72 °C for 2 min (30 cycles).

-Third step: final extension at 72 °C for 7 min marking the end of the amplification reaction. The size of the amplicons were determined by electrophoresis on a 1.5% agarose gel containing ethidium bromide (BET). Amplicons were stored at -20 °C for further use.

#### Restriction analysis

Digestion of the amplicons was performed using the restriction enzymes *Hae III* (*Haemophilus aegyptius*) and *Hinf I* (*Haemophilus influenzae*) reported in the literature [43, 44]. It was performed in Eppendorf tubes containing 20 µL of the reaction mixture. The Eppendorf tubes with amplicons were incubated in a dry water bath (Biobase, China) at 37 °C for 6 h. The sizes of digested fragments were analyzed on 3% agarose gel containing ethidium bromide (BET).

#### Yeast identification by sequencing and sequence analysis

The method used for sequencing was the same that was described by N’guessan et al. [14]. All amplicons digested used to carry out the sequencing. For sequencing, the primers used (NL1 5’-GCATATCAATAAGCGGAGGAAAAG-3’ and NL4 5’-GGTCCGTGTTTCAAGACGG 3’) have been described by Kurtzman and Robnett [45]. The PCR mix was prepared in the same conditions as for amplification of the ITS region. PCR was carried out in 5 steps: firstly an initial denaturation at 94 °C for 5 min followed by 30 cycles of denaturation at 94 °C for 30 s, then primers annealing at 54 °C for 40 s and DNA extension at 72 °C for 1 min 30 s. The last step which was the final extension was completed at 72 °C for 7 min.

PCR amplification and sequencing of the ACT1 gene and the ACT1 gene intron was carried out according to the method described by Jacques et al. [46]. The sequencing of amplified fragments was performed by the company Eurofins MWG Operon (Ebersberg, Germany). The sequences obtained were compared by using basic local alignment search tool (BLASTN) to provide sequence similarity with the sequences available in the National Center for Biotechnology Information (NCBI) GenBank databases (http://www.ncbi.nlm.nih.gov). A multiple sequence alignment was performed, and the phylogenetic tree was constructed by the neighbor-joining method [32] using MEGA (Molecular Evolution Genetic Analysis) software, version X [33].

### Fermentation characteristics of identified yeasts species: *Issatchenkia orientalis* and *Pichia kudriavzevii*

#### Growth at different pH

Antunovics et al. [47] developed a modified approach to assess the growth of the identified species at different pH (3, 4, 5, 6). YPD broth (10 g/L yeast extract, 10 g/L bactopeptone, 10 g/L D-glucose) was previously used to culture the strains at 25 °C. Each specie was then inoculated into 50 mL of YPD broth at 0.2 OD. The culture flasks were then incubated for 24 h with agitation at 150 rpm. After 24 h, the optical densities (OD) were measured at 600 nm with a clear spectrophotometer view UV translluminator/visible spectrophotometer, and the growth of each species was assessed. The experiments were replicated three times.

#### Growth at different temperatures

The growth at different temperatures was carried out according the method described by Antunovics et al. [47]. Samples of overnight cultures (grown at 25 °C) were inoculated into broth YPD broth at O.D. 0.2 and incubated in a water-bath shaker at 30, 40, and 50 °C for 24 h for comparison of growth at different temperatures. The optical density at 600 nm was used to monitor the increase in cell population. The experiments were replicated three times.

#### Analysis of Issatchenkia orientalis and Pichia kudriavzevii resistance to ethanol

The yeast strains were tested for their capacity to grow under ethanol stress using a slightly modified method described by Carrasco et al. [48]. The strains were transferred from Sabouraud-chloramphenicol plates to a pre-culture medium of 10 ml YPD broth (10 g/L yeast extract, 10 g/L peptone, 10 g/L D-glucose) and cultured for 24 h at 30 °C. The pre-cultures were used to inoculate 50 mL of YPD broth supplemented with 0%, 5% percent, 10%, and 15% ethanol (v/v) at an initial cell concentration of 0.2 OD600nm. The cultures were incubated at 30 °C with rotation at 150 rpm for 24 h and tested for vitality at the end of the incubation period. The experiments were replicated three times.

### Traditional sorghum beer production from Issatchenkia orientalis and Saccharomyces cerevisiae

#### Starter preparation

A dense suspension of each specie *Issatchenkia orientalis* and *Saccharomyces cerevisiae* from a Sabouraud-Chloramphenicol plate was prepared in 4 ml of sterile distilled water using a loop. The suspension was mixed with 40 mL of sterile sorghum wort taken from a traditional brewer in Williamsville Macaci (district of Abidjan). Each starter inoculum was incubated at 30 °C for 24 h. The *S. cerevisiae* F12-7 yeast strain was employed was from the culture collection of the Food Technology Department of University Nangui-Abrogoua, Abidjan, Côte d'Ivoire. It was isolated from sorghum beer from the Abidjan district (Southern Côte d'Ivoire). Polymerase Chain Reaction-Restriction Fragment Length polymorphism (PCR–RFLP) of the Internal Transcribed Spacer (ITS) region and sequencing of the 26S rRNA gene's D1/D2 domains were used for its identification [14]. The yeast strain was stored at 20 °C in 20% glycerol.

#### Alcoholic fermentation

Fermentations were carried out in 500 mL Erlenmeyer flasks containing 400 mL sterile wort and closed with a cotton cap. The flasks were inoculated with each specie (*Issatchenkia orientalis* and *Saccharomyces cerevisiae*) at O.D. 0.5 and shaken at 120 rpm for 16 h at 30 °C. The fermentations were carried out three times.

### Analytical Assays

#### Physicochemical analysis

Specific gravity, pH, titratable acidity (TA), total solids soluble (TSS), alcohol rate have been determined as previously described.

#### Organic acids analysis

Organic acids (tannic acid, citric acid, fumaric acid, acetic acid, tartric acid, and lactic acid) were separated and quantified using an HPLC system (Shimadzu Corporation, Japan) fitted with a pump (Shimadzu LC-6A Liquid Chromatograph), a detector (Shimadzu SPD-6A UV Spectrophotometric detector), and an Integrator (Shimadzu C-R 6A Chromatopac). An ion-exclusion ORH-801 column was used for chromatographic separation (300 mm × 6.5 mm, Interchrom, France). The eluant was 0.004 N H_2_SO_4_ at a flow rate of 0.8 mL/min, with a 210 nm detector. For HPLC samples, a 20 µL injection volume was used. The analysis was carried out twice and the mean values were used. The standards for organic acids were dissolved.

#### Statistical analysis

Statistical analysis was used to process the data obtained. Analysis of variance (ANOVA) was performed using XLStat software (version 2016). Mean values physicochemical parameters of fermenting wort and beers were analyzed with Duncan and Tukey’s tests. Differences were considered significant for values of *P* < 0.05. Principal component analysis (PCA) was performed using XLSTAT in order to visualize relationships among variables represented by beers compounds.

## Supplementary Information

**Additional file 1.**

## Data Availability

The datasets generated during and/or analysed during the current study are available from the corresponding author on reasonable request. Accession number(s) can be found below: https://submit.ncbi.nlm.nih.gov/subs/?search=SUB11307678, ON229495-ON229497.
