# Supplementary material for: Identification of non-Saccharomyces yeast strains isolated from local traditional sorghum beer produced in Abidjan district (Côte d’Ivoire) and their ability to carry out alcoholic fermentation
Source: BMC Microbiol. 2022 Jun 27;22:165. doi: 10.1186/s12866-022-02560-8 (PMC9235157; doi:10.1186/s12866-022-02560-8)
Supplement: Supplementary file 1 — Additional file 1. [file 12866_2022_2560_MOESM1_ESM.pdf]

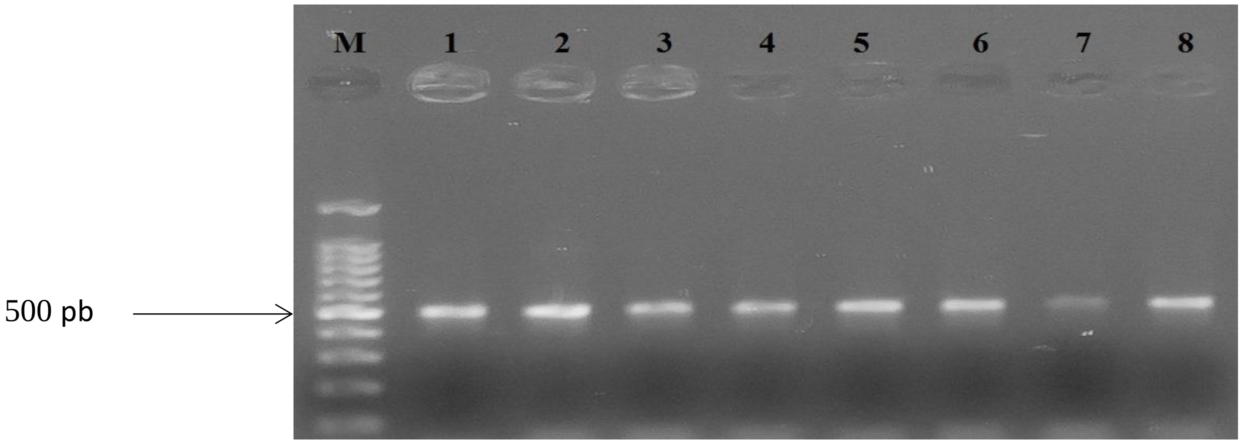

PCR products of amplification by primers ITS1 and ITS4 of 5.8S-ITS region of the rDNA:

M : DNA ladder marker (100 pb); 1-8: NS amplicons

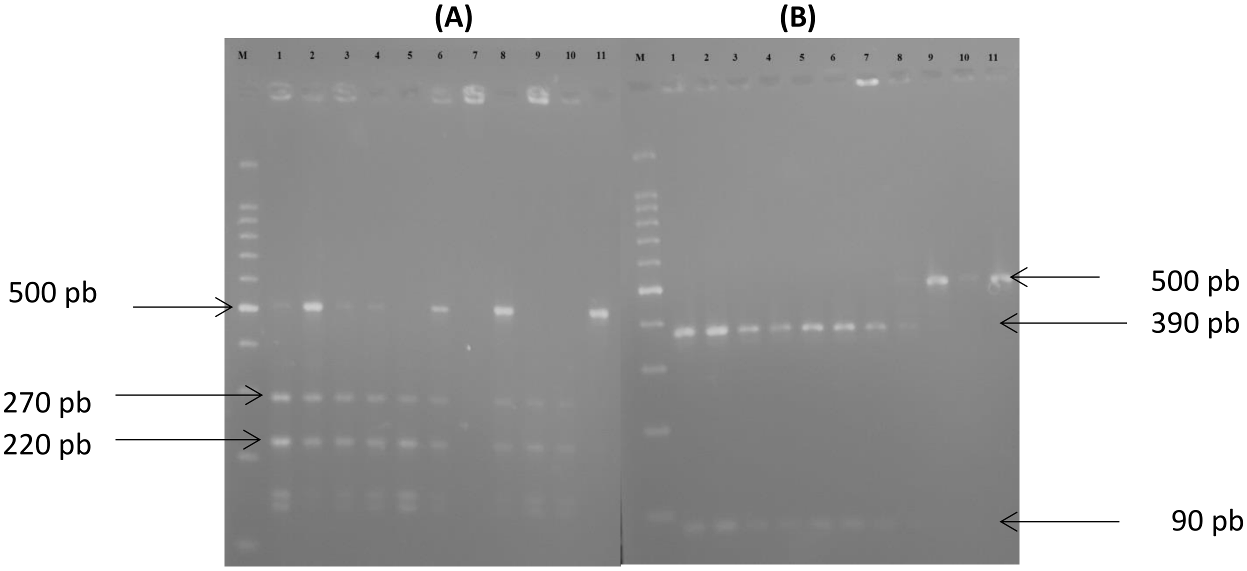

Electrophoretic profile of the restriction fragments of the different amplicons after enzymatic hydrolysis by *Hinf I* (A) and *Hae III* (B)
